# Nails: The Window to the Nose? Update on Yellow Nail Syndrome

**DOI:** 10.5826/dpc.1002a31

**Published:** 2020-04-03

**Authors:** Laura Vollono, Marco Adriano Chessa, Antonio Bruno, Michela Starace, Aurora Alessandrini, Bianca Maria Piraccini

**Affiliations:** 1 Dermatology Unit, Department of Medicina dei Sistemi, Tor Vergata University, Rome, Italy; 2 Dermatology Unit, Department of Experimental, Diagnostic and Specialty Medicine, University of Bologna, Italy; 3 Radiology Unit, Department of Experimental, Diagnostic and Specialty Medicine, University of Bologna, Italy

**Keywords:** yellow nail syndrome, lichen planus, arrested nail growth, respiratory disease

## Abstract

**Background:**

Yellow nail syndrome is a rare condition characterized by typical nail alterations and variable presence of lymphedema and respiratory disease. The pathogenesis is still obscure, with most of the literature deriving from case reports and few investigations. The most reported respiratory conditions associated with yellow nail syndrome are pleural effusion and bronchiectasis, whereas association with rhinosinusitis is rarer.

**Objectives:**

To describe a case of yellow nail syndrome and to provide a literature review regarding this disorder, discussing pathogenetic hypothesis, associated conditions, and therapeutic options.

**Patients/Methods:**

A 49-year-old man presented with arrested growth and alterations of his nails, without any history of previous trauma or inflammation but with a severe nasal septum deviation and a history of chronic rhinosinusitis. A diagnosis of yellow nail syndrome was made.

**Results:**

Six months after undergoing rhinoseptoplasty and treatment with oral vitamin E, the patient’s nails were cured.

**Conclusions:**

This case emphasizes the role of the dermatologist in detecting systemic conditions. The correct diagnosis led to complete resolution of both nail alterations and associated respiratory disorders.

## Introduction

Yellow nail syndrome (YNS) is a rare disorder characterized by typical nail alterations variably associated with lymphedema and respiratory disease. It mainly affects individuals over 50 years of age, although rare pediatric cases have been reported [1–14].

Nail discoloration (chromonychia) varies from pale yellow to dark greenish, with enhanced transverse curvature and onycholysis. The nail plate is thickened, making the nails very hard and difficult to trim (scleronychia) and the lunula is obscured by nail hyperkeratosis. Nail growth rate is half that of normal nails, with disappearing of cuticles. Differential diagnosis includes onychomycosis, drug-induced nail discoloration, trauma, chronic paronychia, and acquired pachyonychia [15].

The pathogenesis is still obscure, with most of the literature deriving from case reports and few investigations. The most common lung conditions associated with YNS are pleural effusion and bronchiectasis, although other respiratory disorders such as recurrent pneumonias, bronchitis, and chronic sinusitis have been included as diagnostic criteria [2,3]. The course of the nail disease does not necessarily parallel that of the associated conditions. We hereby report a case of YNS with severe nasal septum deviation and chronic rhinosinusitis that completely resolved after surgical treatment of the respiratory disease and oral administration of vitamin E, together with a comprehensive review of the literature regarding this peculiar entity.

## Case Report

**Figure 1:**
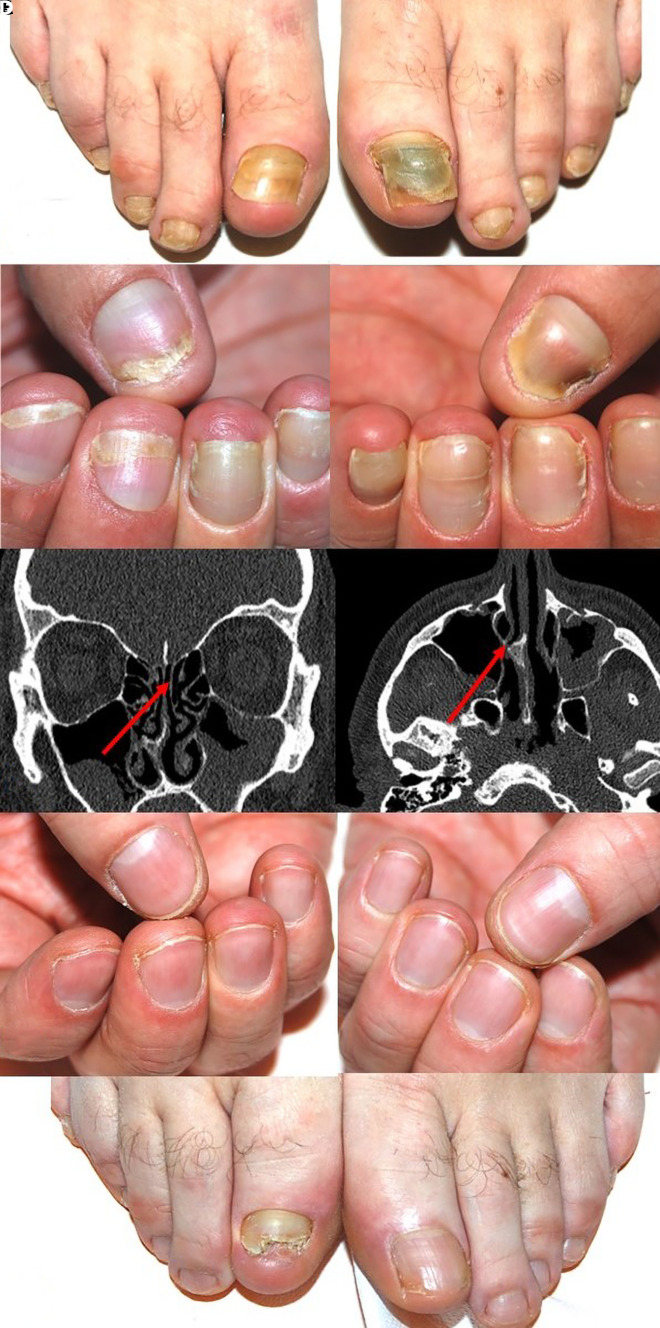
Nail alterations characteristic of yellow nail syndrome. (A,B) Absence of the cuticles and nail fold swelling are key to diagnosis. (C) Head CT scan revealing type II septal deviation (Mladina classification) associated with unilateral spur compromising the airway (arrow, left) and secondary hypoplasia of the right middle nasal turbinate (arrow, right). (D,E) Improved appearance of the nails 1 year after rhinoseptoplasty and oral vitamin E supplementation.

A 49-year-old man presented to our Nail Clinic with arrested growth rate and alterations of his nails that started 6 months prior. His medical history was unremarkable, except for chronic rhinosinusitis. Dermatological examination revealed increased transverse curvature, onycholysis, yellow-green discoloration, and nail fold swelling. Notably, the cuticles were lacking (Figure 1, A and B). He denied any previous trauma or manipulation of his nails. Routine nail fungal culture was negative. Neither swelling of the limbs nor facial edema was observed. Based on the clinical picture and medical history, a head CT scan was performed, which detected a severe nasal septum deviation compromising the airway (Figure 1C). Chest x-ray was unrevealing. A diagnosis of YNS with chronic rhinosinusitis was made. The patient underwent rhinoseptoplasty and treatment with oral vitamin E (1,200 IU/day). One year later, his respiratory symptoms had resolved and his nails appeared completely cured (Figure 1, D and E).

## Discussion

YNS was first described in 1964 by Samman and White, who reported the association between typical nail findings and lymphedema [16]. Two years later, Emerson added pleural effusion as a further diagnostic criterion [17]. Later on, other chronic respiratory symptoms including sinusitis, bronchitis, recurrent pneumonias, pleuritis, and bronchiectasias have been included in the characteristic pulmonary manifestations of YNS [16–20]. Only 2 criteria out of 3 are required to diagnose YNS, and the complete triad is reported to be present in only 27%–60% of cases [1–3,16,17,19–22]. The possible interval between the first clinical sign (lymphedema, lung manifestations) and nail alterations hinders affirmation of the diagnosis; thus the condition may be underestimated.

### Associated Conditions

The occurrence of lymphedema ranges between 29% and 80%, being the first sign of the syndrome in approximately one-third of cases [2,3,19]. Clinical characteristics are the same as those of primary lymphedema, most commonly occurring on lower limbs, although facial edema and upper limb lymphedema have been rarely reported [16,23]. Prevalence of pleural effusions is approximately 14%–16% in YNS, with the prevalent clinical manifestation being chronic cough, and bronchiectasias are present in approximately 44% of patients [2]. Acute or chronic sinusitis is frequently observed (14%–83%), mainly affecting the maxillary and ethmoid sinus [1–3,19,21,22]. CT scans usually show mucosal thickening with enlargement of turbinates and fluid levels.

YNS has been also associated with several systemic conditions. Hydrops fetalis, primary intestinal lymphangiectasis, and lymphedema-distichiasis syndrome has been reported in patients with YNS, suggesting lymphatic impairment as a key factor in the development of the syndrome [24–26]. Cases of iatrogenic YNS following cardiac mitral valve replacement, implantation of permanent titanium cardiac pacemaker, and coronary artery bypass grafting have been recently published [27–29]. Autoimmune diseases (primarily rheumatoid arthritis) and immune deficiency have been occasionally associated with YNS, partially explaining the proneness to chronic infection of the respiratory system [30–33]. The hypothesis that YNS may be a paraneoplastic syndrome derives from reports of malignant diseases diagnosed concurrently or closely thereafter in YNS patients [2,3,17,34–42]. The frequency of association between YNS and various types of cancer is low and the matter remains controversial; however, a careful anamnesis of medical history and possible signs of systemic disease is recommended.

### Pathogenesis

The etiology of YNS is obscure and investigations are few. Most of the literature is from case reports, and the majority of case series include 3 or fewer patients, although large series of up to 41 cases have been published [1–3,21,23]. Despite the paucity of evidence, a unifying lymphatic mechanism has been proposed to explain the development of lymphedema, pleural effusion, and nail alterations in YNS. Defective lymphatic drainage may be responsible for the sclerosis of the nail matrix tissue observed at light microscopy examination, with ectatic, endothelium-lined channels embedded in the fibrotic stroma, resulting in the clinically evident slow growth and thickening of the nails. The same mechanisms could be held responsible for the sclerosis and dilated lymphatic vessels also found in parietal pleura and subcutaneous tissue of YNS patients.

In the largest series published to date, Maldonado et al hypothesized that YNS pathogenesis might be due to microvasculopathy associated with protein leakage, rather than lymphatic functional impairment [2]. However, a recent case-control study on 17 YNS patients with lower and/or upper limb lymphedema found a significantly higher rate of lymphatic morphological abnormality and reduced regional nodal uptake compared to healthy controls, concluding that YNS should be considered as a lymphatic phenotype [23]. As suggested, it seems likely that one given unknown factor, genetic and/or extrinsic, determines a decline in lymph vessel function, leading to peripheral sclerosis of subcutaneous tissues and morphological abnormality of lymph vessels that worsen as the YNS clinical manifestations become more evident. The role of titanium, especially titanium dioxide, has been recently evoked, although the mere exposure to the agent does not seem sufficient to develop the syndrome [43–45]. In any case, a detailed exposure history from patients with YNS is indeed recommended.

### Treatment

A careful review of the literature points out that there is no consensus regarding YNS treatment, owing to the lack of large-scale studies. Treatment is typically based on anecdotal evidence, case reports, or small series. Spontaneous resolution without any treatment has been observed. Cancer treatment showed to lead to resolution of symptoms in patients affected by paraneoplastic YNS [12]. Topical treatments for nail changes such as triamcinolone injections and topical α-tocopherol (vitamin E) have been proposed, with inconsistent results [46–50]. Management of lymphedema with manual lymphatic drainage and compression bandages is advisable in order to prevent irreversible hypertrophy of subcutaneous tissue [19].

Regarding systemic treatment, oral zinc sulfate supplementation (300 mg/day) resulted in improvement of lymphedema and nail alterations, but no effect on pulmonary manifestations was observed [51]. Triazole antifungals such as itraconazole showed to exert limited effects on affected nails; however, combination treatment with pulsed oral fluconazole and oral α-tocopherol resulted in significant improvement of nail alterations [3,52–55]. The therapeutic effect may be exerted by a combination of the azole antifungals’ stimulation of linear nail growth and the vitamin E antioxidant properties that prevent colorless lipid precursor to be transformed into lipofuscin pigment responsible of nail yellowing [54,56,57]. Oral vitamin E is traditionally prescribed also as monotherapy. Because of its efficacy for chronic lower respiratory tract infections, clarithromycin (CAM) theoretically makes a suitable candidate as therapeutic agent in YNS. Apart from a single case report in 2011, a recent observational study on 5 YNS subjects with respiratory manifestations reported significant improvement of nail discoloration in parallel with improvement of respiratory symptoms after oral administration of CAM [58,59]. The initial dosage was 200 mg/day, but therapeutic effects were not observed until the dosage was increased to 400 mg/day, suggesting that the activity of CAM in YNS may be dose-dependent. The therapeutic effect may be exerted by both antibacterial and anti-inflammatory activity of CAM, improving both lymphatic drainage around nails with resolution of discoloration and reducing water and mucus secretion in the respiratory tract [60–62].

Treatment for chronic rhinosinusitis is not specific for YNS patients; however, global response to medications such as oral antibiotics or topical intranasal steroids or decongestants is poor, making surgical procedures often necessary. A few cases of YNS with sinobronchial syndrome in which treatment with CAM led to resolution of both nail and respiratory manifestations have been reported [58,59]. A case of a patient affected by YNS with rhinosinusitis cured with combination treatment with triamcinolone injection, oral vitamin E, oral fluconazole, and robust medical regimen for rhinosinusitis has been reported, although the variety of treatment administered makes it is difficult to assess the relevance of each agent in the resolution of symptoms [50]. A single case of YNS with rhinosinusitis whose nail manifestations were dramatically improved after endoscopic sinus surgery has been reported [63].

## Conclusions

To our knowledge, this is the fourth report regarding successful treatment of YNS manifestations in a patient with chronic rhinosinusitis, and the second one observing resolution of YNS symptoms after sinus surgery. Notably, our patient underwent combined treatment with both rhinoseptoplasty and oral vitamin E supplementation, suggesting that a multidisciplinary therapeutic approach may be more likely to lead to a cure than a single medicine or treatment.

Further research is indeed required in order to investigate the potential of the above-mentioned therapeutic strategies and provide higher levels of evidence for the treatment of YNS. This report aims to emphasize the role of the dermatologist in detecting systemic conditions, sometimes hidden behind what seems to be a mere aesthetic concern.

We believe that our case is of special interest as the correct diagnosis led to appropriate therapeutic strategies, resulting in the complete resolution of both the nail alterations and associated respiratory condition. Careful anamnesis regarding medical history and associated conditions should always be performed in order not to miss an opportunity to provide patients with proper care.
